# Implementation of a software defined FLISR solution on an active distribution grid

**DOI:** 10.12688/openreseurope.14115.2

**Published:** 2022-09-20

**Authors:** Darren Leniston, David Ryan, Clem Power, Paul Hayes, Steven Davy

**Affiliations:** 1 Programmable Autonomous Systems Unit, Walton Institute for Information and Communication Systems Science, Waterford City, Waterford City, Co. Waterford, Ireland; 2 Electrification Innovation & Delivery Network Assets, ESB Networks, Rathnapish, Co. Carlow, Ireland; 3 Electrification Innovation & Delivery Network Assets, ESB Networks, Cork City, Co. Cork, Ireland

**Keywords:** FLISR, Smart Grids, Self-Healing, Distribution Automation, SCADA

## Abstract

Background: The resiliency of the distribution grid is of increasing concern to the distribution system operator (DSO) due to factors such as climate change and the resulting faults caused by inclement weather conditions, leading to service disruption to consumers. Loss of service negatively affects key performance indicators (KPI) of the DSO, such as customer minutes lost (CML) and customer interruptions (CI), leading to financial penalties imposed by the regulator.

Methods: In this paper we propose a software-driven Fault Location, Isolation and Service Restoration (FLISR) solution, leveraging modern software and communication technologies married with the DSO's existing infrastructure, to aid fault detection and resolution, with the aim of reducing CMLs & CIs and curtailing the financial penalties incurred.

Results: The proposed FLISR solution was trialled in an area of south-east Ireland which sees a higher count of service loss as compared to more inland areas, providing an ideal environment to gauge the effectiveness of the solution. It was found that the solution generated outputs that could potentially lead to the resolution of fault events faster than the current systems in place by the DSO.

Conclusions: Based on the results gathered from operating the FLISR solution on an active grid, it has demonstrated that leveraging modern software technologies in tandem with existing grid infrastructure benefits the DSO with reference to grid management and operations and the customer in terms of quality of service site.

## Nomenclature

**Table:** 

ASDU	Application Service Data Unit
CHL	Customer Hours Lost
CI	Customer Interruptions
CML	Customer Minutes Lost
CRO	Control Room Operator
DSO	Distribution Grid Operator
FLISR	Fault Location, Isolation and Service Restoration
FPI	Fault Passage Indicator
ICT	Information Communication Technology
IEC	International Electrotechnical Commission
IOA	Information Object Address
IoT	Internet of Things
kV	Kilovolt
LBFM	Load Break Fault Make
LVI	Loss of Voltage Indicator
MQTT	Message Queuing Telemetry Transport
MV	Medium Voltage
N/O	Normally Open
NDCC	National Distribution Control Centre
RES	Renewable Energy Sources
RTU	Remote Terminal Unit
SCADA	Supervisory Control and Data Acquisition

## Introduction

In an era where climate change is being linked to more frequent and powerful storms, the need for resilient power systems is growing ^1^. In recent years the energy sector has been going through a transformation from a hardware to software orientated focus with the emergence of Internet of Things (IoT) technologies ^2^ and Artificial Intelligence. This is leading to large bodies of research, with an aim to increase grid resiliency, cater for the integration of renewable energy resources (RES), and exploit data gathered from the electrical network to provide analysis techniques that can detect faults on the system and aid in restoring power in a way that will impact fewer consumers. The motivation for this research is centred on economic and quality of service grounds, focusing on how the restoration of supply to as many consumers in the shortest time possible obviously benefits the consumer, but also saves the distribution system operator (DSO) from the imposition of financial penalties for customer minutes lost (CMLs). FLISR is one such technique, and in this paper we present a software based approach that utilises grid models coupled with real-time data gathered from certain existing pole-mounted grid components, namely electrically and remotely operable switches & reclosers. It is for this reason the proposed solution is described as “software defined”, as it utilises communication and data streams from devices already present on the grid with no requirement for new hardware to be installed. The following sections frame the research, expand on the motivation and background work that formed the basis of the software implementation, and how the proposed technique levers existing data sources and hardware components. The results are presented, followed by the future work and conclusion where the potential of the research in the DSOs operations is discussed with the next steps needed to realise the potential of the proposed solution. The requirement for a more reliable grid has led to the exploration and implementation of FLISR by utilities to meet this goal. A Department of Energy report in 2014 conducted a study of five implementations of FLISR and concluded a reduction of CMLs and CIs by up to 51% and 45% respectively for a fault event leading to outage ^3^. From the literature, there are various implementations of the FLISR process, from locating a fault through methods such as apparent impedance ^4^ and travelling waves ^5^ or through power quality measurements. Similarly, the generation of optimal restoration steps can be undertaken through a variety of approaches. Eriksson *et al.* ^6^ propose a multiagent FLISR algorithm utilising Prim’s Algorithm. Kock-Ciobotaru *et al*. ^7^ describe a FLISR implantation through the use of IEC 61850 GOOSE with the algorithm operating as a finite-state machine. Shahsavari *et al.* ^8^ propose a healer reinforcement approach to a FLISR implementation. In reviewing the literature, it is clear there is a requirement to move towards more advanced, distributed methods, which apply modern standards and ICT to provide higher reliability of the grid.

The FLISR solution described in this paper was tested on sections of the distribution grid located in key locations in Ireland, where the effect of adverse weather conditions on exposed and vulnerable networks causes higher occurrences of loss of supply to the consumer. The trial sites in question employ grid resilience strategies on the medium voltage (MV) network to curtail the effect of faults on consumers and the financial penalties imposed on the DSO, these strategies take the form of manual and automated schemes operated by pole-mounted switches and reclosers.

While these strategies go some way to reducing the impact of faults, they possess certain limitations. For example, the pole-mounted switches are load break fault make (LBFM) remotely operable switches, and as such they do not open/close autonomously. The switches communicate with the DSOs supervisory control and data acquisition (SCADA) system, assisting control room operators (CRO) in identifying the faulted section of network. Subsequently, the manual opening and closing operations are performed remotely from the National Distribution Control Centre to sectionalize and restore supply. This requirement for manual intervention on the part of the CRO to operate the switches introduces bottlenecks in the speed of fault resolution and ultimately increases the cost to the DSO. The introduction of loop automation through pole-mounted reclosers goes some way in mitigating the efficiency concerns observed with manual intervention. Within this configuration, reclosers operate as their own autonomous islands with no requirement for intervention from the central control room. In the event of a fault, reclosers operate autonomously based on timers and loss of voltage ^9^. This approach has some disadvantages however, as the faulted section can at times be re-energised by alternative supply before it is isolated from the network. Additionally, the pole-mounted reclosers only operate autonomously when part of a loop automation scheme. In relation to the Irish distribution grid, the DSO observed that earlier versions of pole-mounted reclosers required re-commissioning, as their firmware did not originally support loop automation functionality, thus leading to a large financial investment. Though the autonomous nature of this scheme resembles some characteristics of self-healing, it does not meet the requirements of the self-healing concept. In relation to the smart grid, self-healing describes the capacity for the systems existent on the distribution network to act autonomously to restore themselves in the event of a sustained or permanent fault ^10^ and such systems should leverage information and communications technology (ICT) to enable monitoring, control and data management ^11^. These factors provided the motivation to explore the development and implementation of an autonomous, software-driven solution for the Irish distribution grid. The solution applies the concept of FLISR ^6^ and is supported by back-bone services to collect and analyse data streams from pole-mounted devices in the field. The goal of the FLISR concept is to optimally restore supply to the largest number of consumers as possible, by intelligently and autonomously isolating and reconfiguring the affected parts of distribution grid ^12^.

In this way, the FLISR solution described in this paper utilises elements of the self-healing concept through the usage of ICT technologies to enable system awareness and autonomous generation of restoration steps. However, it should be noted that this solution could be described more accurately as “impact-mitigation” rather than full self-healing, due to the need for network operators and engineers to manually repair physical damage to the grid as a consequence of fault events caused by storms and other inclement weather conditions.

## Methods

### Selecting a trial site

**Figure 1.**
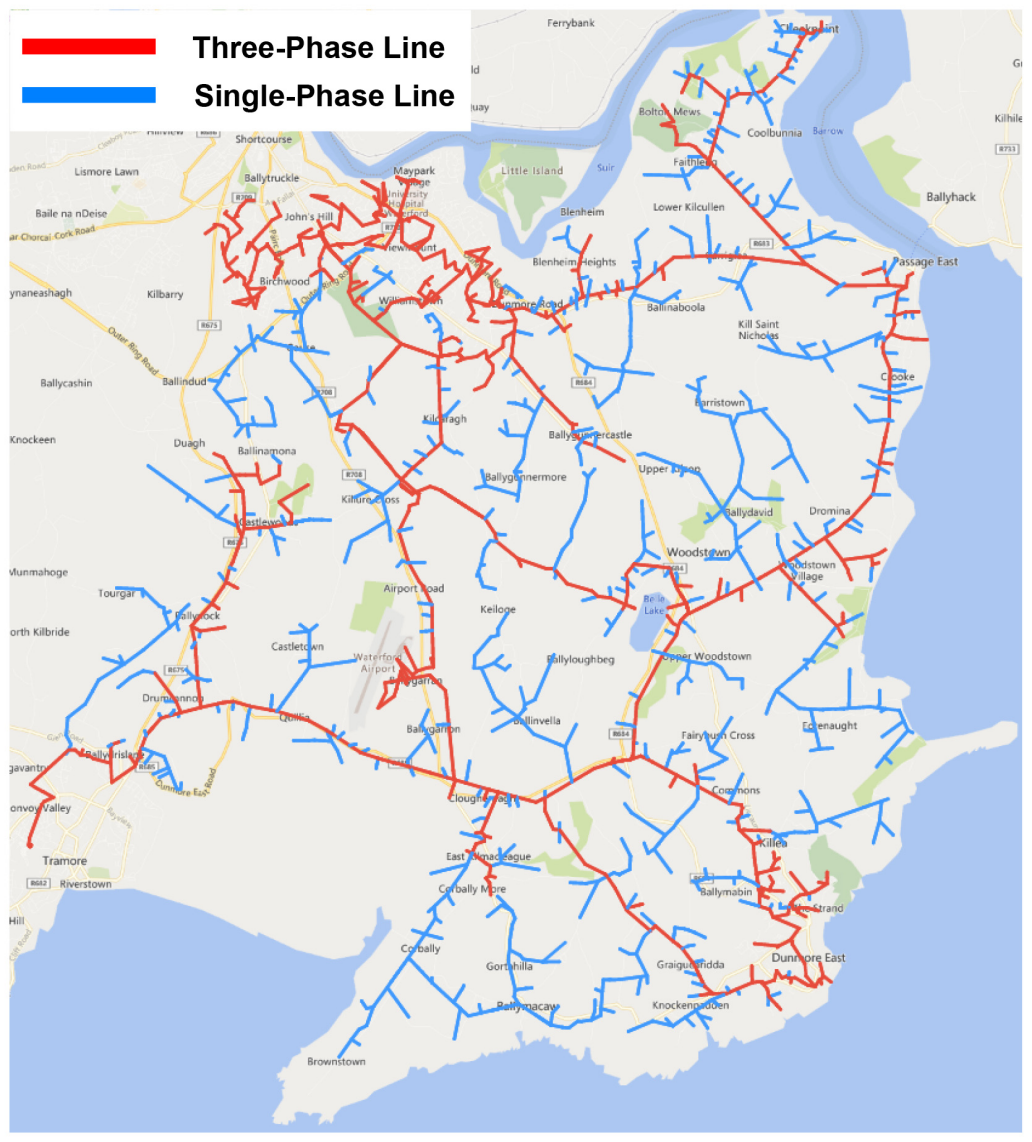
Trial site distribution grid.

The implementation of the proposed FLISR system commenced with devising a series of fault restoration steps for a portion of the distribution grid located in the south-east of Ireland. The site was selected for its proximity to the coast resulting in a higher occurrence of fault events and service disruption compared to inland areas ^13^, its structure as a complex radially fed network of single and three phase lines, shown in Figure 1, and a lack of an existing loop automation scheme. 

**Figure 2.**
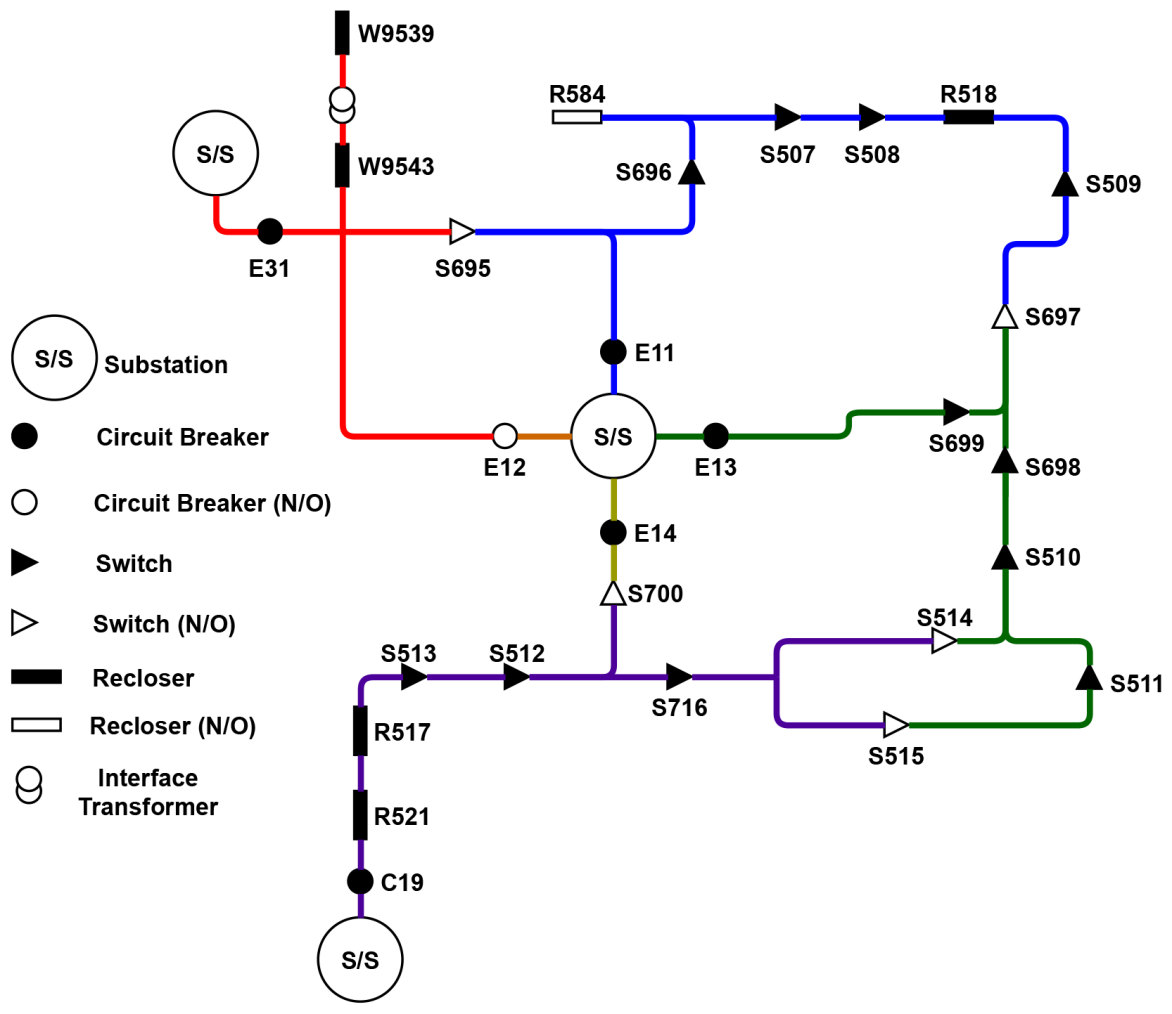
Trial site circuitry.

**Figure 3.**
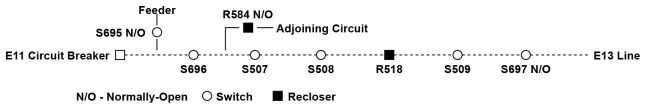
Subsection circuit.

The trial site network is divided into four primary sections, supplied by one of three 38kV substations with a total of five MV feeders. Each section contains a number of assets, including pole-mounted switches and reclosers that are identifiable through a unique SCADA ID, with select devices designated as normally-open (N/O) points to facilitate interconnection to adjoining outlets. Figure 2 describes in more detail the circuitry of the trial site distribution grid. A subsection of this network was selected as a starting point for developing the FLISR solution, presented in Figure 3.

The selected subsection E11 is fed supply from a 38kV substation, with connection to an adjoining circuit and sections E13 and E31 through interconnecting normally-open devices, providing a source of back-fed supply if necessary ^14^. Pole-mounted devices on the line communicate two key data points in the event of a fault; a fault passage indicator (FPI) and a loss of voltage indicator (LVI) ^15^, which are used by the CRO to determine the approximate location of the fault, after which the faulted section is isolated and supply is restored from an adjoining section or circuit through closing a normally-open device. The DSO used their in-house expertise to investigate the E11 line and determine all permutations of fault events that could occur, and the effective resolution steps for each, and these were correlated into "fault cases" as presented in Table 1.

**Table 1.** List of fault cases for the E11 line.

Fault case	Fault location	Recommendation
0	No fault	No recommendation
1	Between E11, S696, S507 and S696	Open S696, Close N/O (S697 or R584)
2	Between S696, S507 and R584	Open S696, S507, Close E11, Close N/O (S697)
3	Between S507 and S508	Open S508, S508, Close E11, Close N/O (S697)
4	Between S508 and R518	Open S508, R518, Close E11, Close N/O (S697)
5	Between R518 and S509	Open S509, Close N/O (S697)
6	Between S509 and S697	Open S509, Close R518
7	Fault detected	Inconclusive reading - investigate further

**Figure 4.**
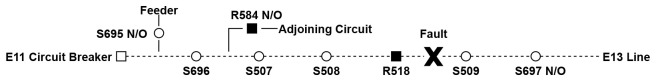
Fault case circuit.

The automatic statuses of the pole-mounted devices were contrasted against recommended operations which were formulated to resolve each of the fault cases in the most efficient manner. Taking the fifth fault case as an example, where a fault has occurred between recloser R518 and switch S509, as illustrated in Figure 4.

The following Table 2 describes the status of each device (FPI and LVI) and its automatic status in the event of a fault before manual intervention, with the recommended operations contrasted.

**Table 2.** Fault case device status and recommended operation.

E11 Line	E11 CB	S695 N/O	S696	R584 N/O	S507	S508	R518	S509	S697 N/O
FPI	false	N/A	false	N/A	false	false	true	false	N/A
LVI	false	false	false	false	false	false	true	true	N/A
Auto status	close	open	close	open	close	close	open	close	open
Recommended ops	close	open	close	open	close	close	open	open	close

**Figure 5.**
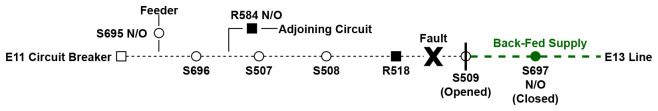
Fault case restoration.

In the above case, the recommended actions result in isolating the fault by opening switch S509 and closing the N/O point, thereby back-feeding from line E13 and restoring supply, as illustrated in Figure 5.

**Figure 6.**
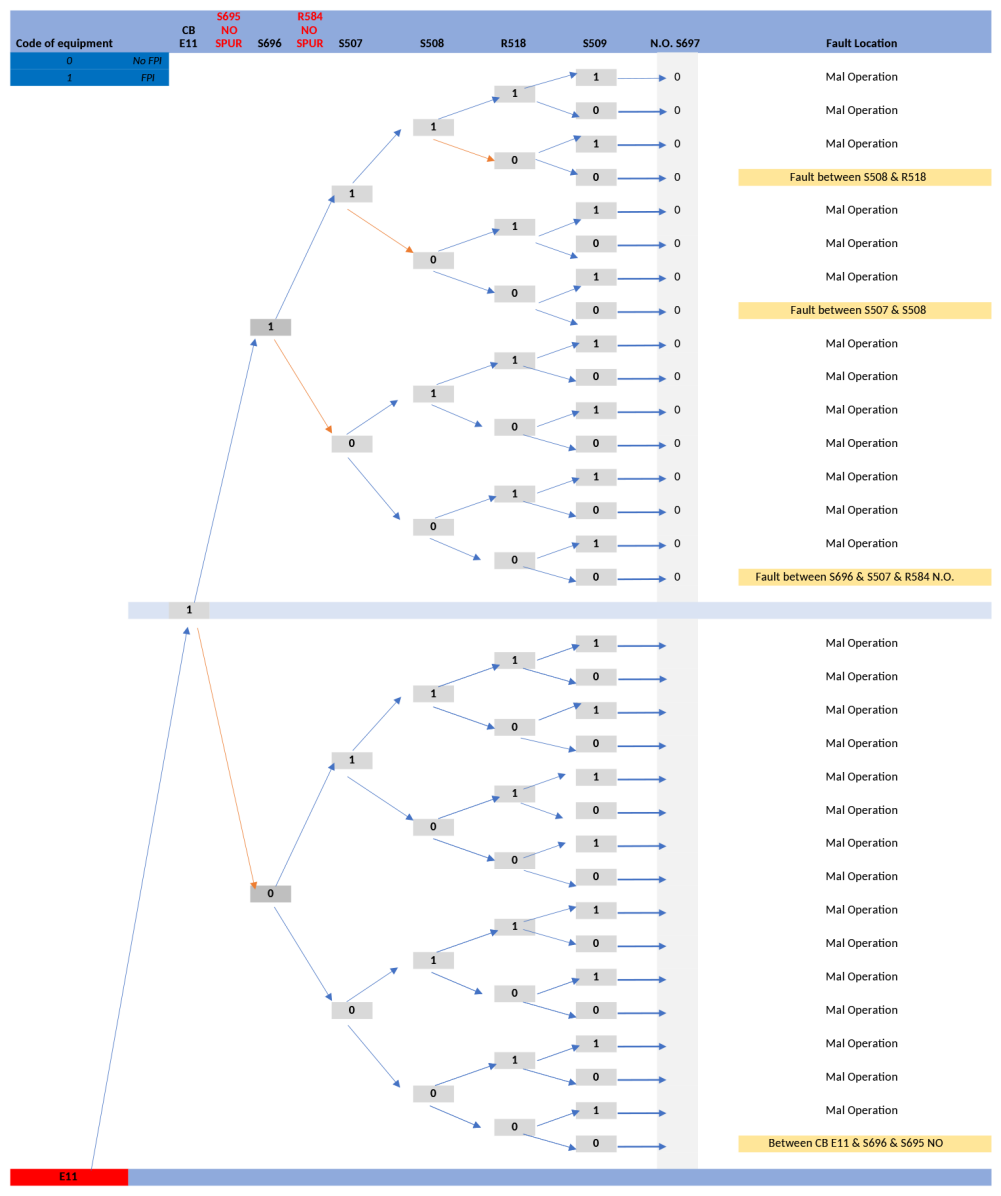
E11 line fault case decision tree.

To aid the development of the FLISR software algorithm, a decision tree was created based on the fault cases described in the above table. This decision tree provided a graphical representation of the processing logic for the fault cases ^16^, a portion of the decision tree is presented in Figure 6.

Both the fault cases and decision tree were assessed to discern the logic by which restoration steps were decided by DSO operation staff, with the aim to translate these into a generalised software algorithm which would be applicable to multiple grid configurations. To evaluate this, the fault cases and restoration steps given by the DSO for the E11 line provided the basis for software tests to validate the FLISR algorithm. Once complete and validated, the FLISR solution algorithm was then applied to all lines of the trial site circuit with the outputs validated by DSO personnel. 

### Application to the field 

The FLISR algorithm developed as an output of the above research requires real-time data from pole-mounted devices that match the FPI and LVI values outlined previously. Switch and recloser devices each have a set of unique data points which correspond to the asset’s current operational status, communicated to the DSO’s SCADA system via a wirelessly enabled Remote Terminal Unit (RTU) employing the International Electrotechnical Commission (IEC) protocol standard, IEC 60870-5-101/104 ^17^. Through this protocol, the data points map to an information object address (IOA), and the devices are identifiable through a unique application service data unit (ASDU) type ^18^. Two data points that match FPI and LVI were identified for both switches and reclosers, described with their corresponding IOA value in Table 3.

**Table 3.** FPI and LVI mapping to switch and recloser devices.

Data point	Switch data	Recloser data
Fault passage indicator	Fault current - IOA 4	End of protection sequence - IOA 27
Loss of voltage indicator	AC supply fail- IOA 2	Controller locked out - IOA 34

The devices communicate operational data when triggered by an event on the distribution grid; in the case of a fault, all affected devices communicate a combination of data points. Devices on the faulted section communicate FPI and LVI, while devices on sections which have lost supply but are otherwise undamaged, communicate LVI. Device data is collected through supplementary communication hardware installed within the existing pole-mounted devices on the trial site grid. This new communication hardware utilises IEC 101 to Message Queuing Telemetry Transport (MQTT) communications, a lightweight messaging protocol suitable for systems with large numbers of IoT enabled assets, due to its speed, scalability and network bandwidth efficiency ^19^.

Device operational data captured from the field is accessible to the FLISR solution and used to generate fault location and restoration outputs. The FLISR solution is also supplied with a grid model of the trial site, containing asset data for pole-mounted devices including SCADA and ASDU identifiers, device type (switch or recloser) and N/O status. As a result, the FLISR solution is aware of each pole-mounted device and its position relative to other devices, thereby achieving a system awareness that did not exist previously.

The FLISR solution software itself is containerised so that it may be deployed within a wide range of computing environments ^20^. In this way, the FLISR solution software offers the flexibility for system operators to deploy in a variety of contexts, for example cloud computing platforms or onto local hardware at the network edge. In addition, the size of the network in which the FLISR solution will monitor will depend on the grid model supplied on deployment. In this way, the solution is scalable through both compatibility with variable size of grid models, and by leveraging the flexibility of its containerised nature by deploying multiple instances of the solution with a unique grid model for each instance.

### FLISR algorithm operation in the field

**Figure 7.**
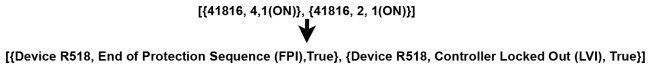
Generated fault case translated.

In operation, the FLISR solution constantly monitors activity from the pole-mounted devices. When a fault occurs on a section, each pole-mounted device affected will communicate their status (FPI and LVI). The FLISR solution uses the detected device’s ASDU identifier, referenced against the grid model, to identify the section in which the fault has occurred. From this point, the FLISR solution retrieves all last available readings from pole-mounted devices on the affected line to build a fault case, which takes the form of a data structure that is parsable by the FLISR algorithm. The fault case contains the faulted device’s ASDU identifier, FPI/LVI IOA address and value. an example for the fifth fault case and the translated meaning is presented in Figure 7.

**Figure 8.**
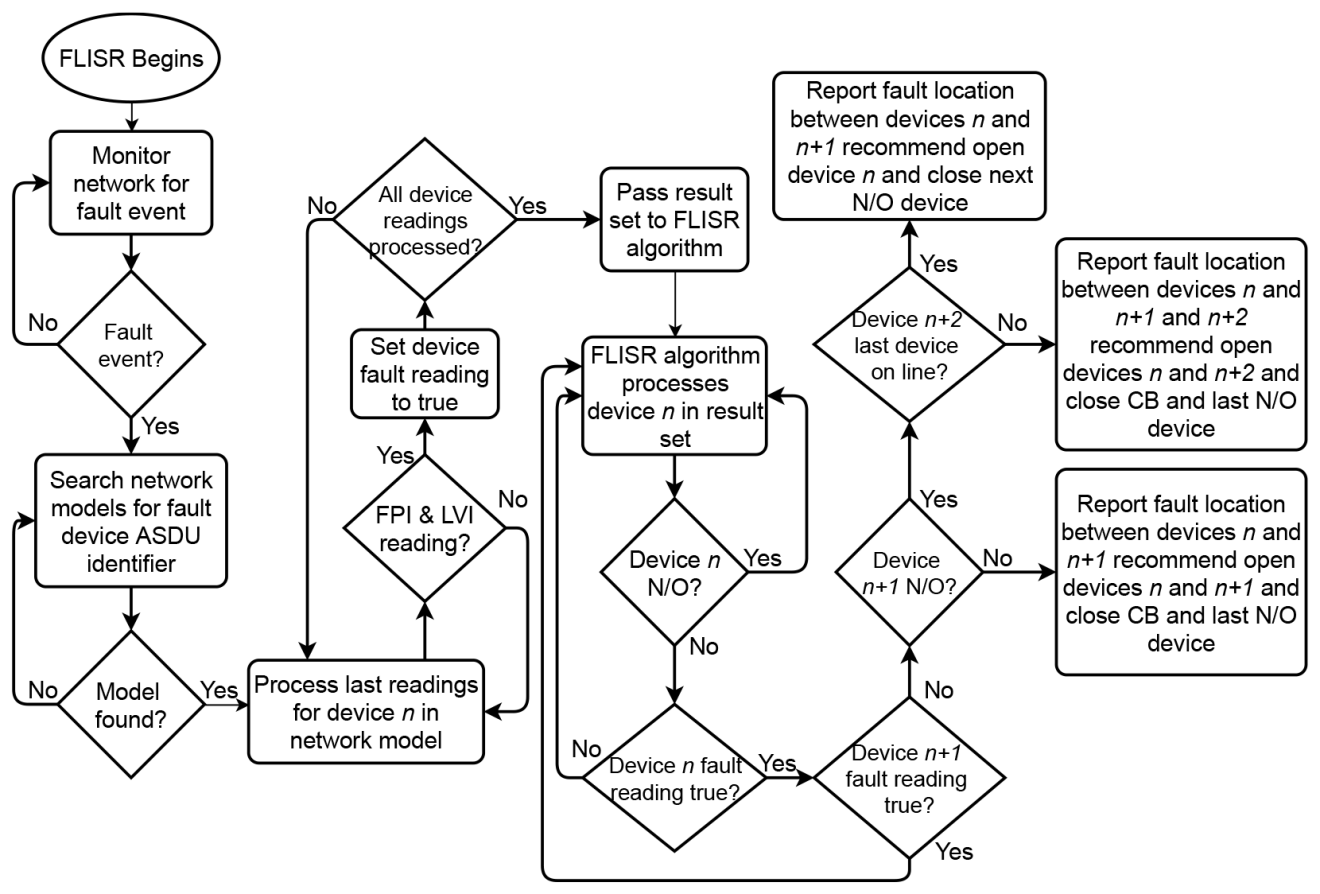
FLISR algorithm operation.

The assembled fault case is processed by the FLISR algorithm. Working backwards from the end of the line, the algorithm locates the faulted section by identifying occurrences of FPI and LVI readings from devices. The FLISR algorithm will then determine recommended restoration steps, suggesting which devices to open in order to isolate the fault, such as the two devices at either end of the fault and, depending on the position of the normally-open points on the line, which devices to close in order to return supply. The algorithm operates in two parts: first, it uses measurement data to create a resulting set of devices in the grid model that communicated a fault reading (FPI and LVI). This result set is then passed to the main FLISR algorithm to determine fault location and recommend the appropriate restoration steps. The algorithms described for the FLISR solution have been developed to be generic so as to apply to any section of network, with the fault cases highlighted previously serving to validate the algorithm outputs. The operation of the FLISR algorithm is illustrated in Figure 8.

### FLISR algorithm output 

**Figure 9.**
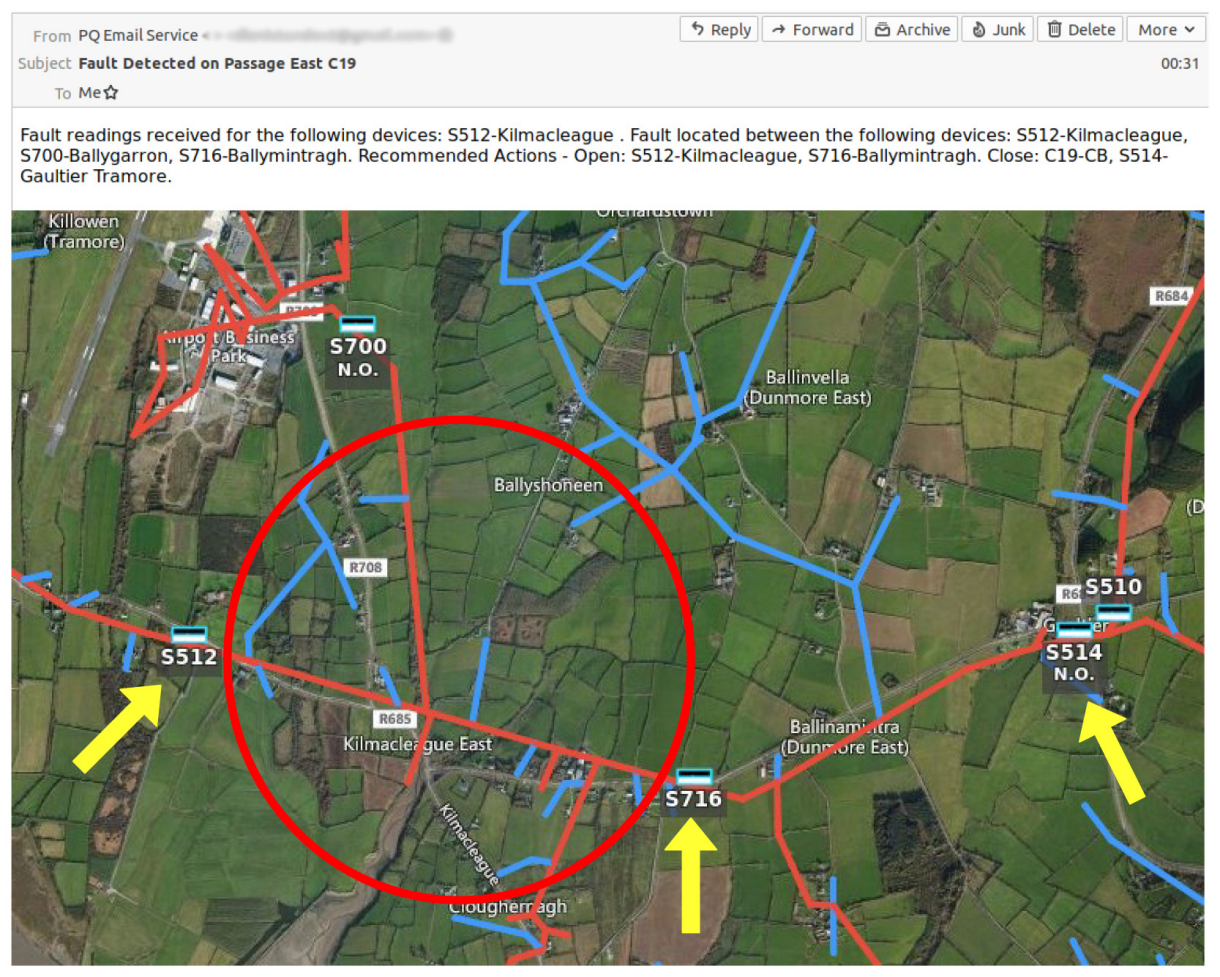
FLISR solution output and location on geographical map.

While it is possible to enable bi-directional communications and enact opening/closing operations on the pole mounted devices through the FLISR solution, to mitigate the DSO’s concerns relating to full automation, this is not enabled. Once the FLISR solution has completed processing a fault event, the DSO’s operations staff is notified via email, reporting details of the faulted devices, the approximate location of the fault as identified by the FLISR algorithm and the recommended restoration steps. This allows for a human operator to be included in the loop and verify that the FLISR solution has recommended reasonable steps to isolate and restore the detected fault, Figure 9 illustrates an email report generated by the FLISR solution in reaction to a fault on the trial site, with the fault location identified highlighted on a geographical map.

## Results

### Results from the trial site

**Figure 10.**
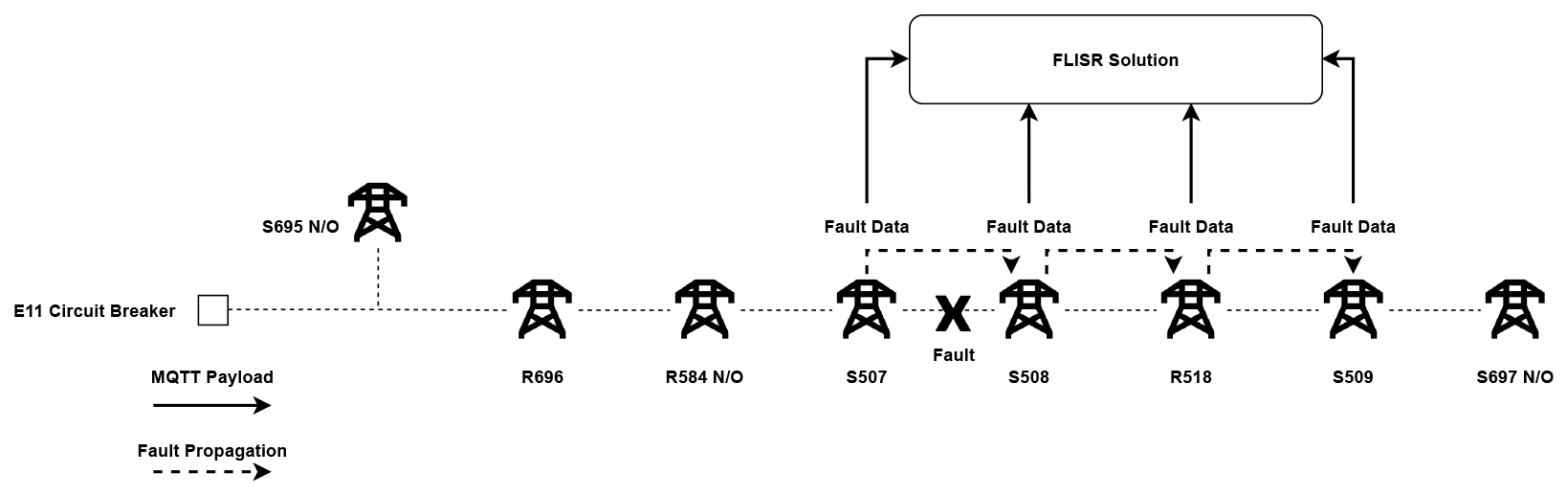
Propagation of fault through a line.

The FLISR solution was deployed to monitor the chosen trial site distribution network over a period of four months, and during this time a number of fault events were captured by the system. However, during the initial operation of the solution, it became clear that fine-tuning of the software algorithm was necessary, as it was found that the system was generating a number of email reports per fault. The reason for this is due to the mechanics of how fault events propagate on the network from their point of origin. In the event that a fault occurs, the pole mounted switch that initially detects the fault communicates a data payload of FPI and/or LVI to both the DSO’s SCADA system and also to the FLISR solution. As the loss of supply is detected along the line by the switches following the faulted section, they in turn also send an individual FPI and/or LVI data payload as illustrated in Figure 10. In theory, each switch should detect and communicate the loss of supply instantaneously, though in practice a very slight delay was observed between data payloads communicated from each faulted switch.

As a consequence, the FLISR solution was detecting each data payload as a separate fault event due to the speed of the solutions operation, therefore generating incorrect results. To mitigate this, the solution was refactored slightly to ensure an email report was generated only after a fault event had completely propagated throughout a line, leading to an increase in the amount of time between the detection of fault event and generating an email report, though only by a fraction of a second. Exploring the results subsequent to the refactor, the average time from fault detection and an email report received fell within a one-minute bracket. Table 4 presents a selection of fault results detected by the FLISR solution, including the time of fault detection and receipt of email report.

**Table 4.** Fault detection and email report timings.

Faulted devices	Fault detected	Email report received
E11 - S508, R518	19/06/2020 - 17:57:17	19/06/2020 - 17:57:29
E11 - R584, R518	25/06/2020 - 11:40:05	25/06/2020 - 11:40:05
C19 - S716	28/06/2020 - 01:11:00	28/06/2020 - 01:11:06
E13 - S699, S698	08/07/2020 - 16:28:56	08/07/2020 - 16:29:14

For this first iteration of the system, the intention is for the generated email report to be forwarded to the DSO’s central control room, whereby a human operator would verify the validity of the report and the fault resolution steps suggested and act on these recommended steps according to their judgement, thereby reducing the amount of time for an operator to manually locate the fault, verify it is a true fault event and finally decide on and enact a set of steps to resolve the fault. From the results presented above, the timing between fault detection and reporting from the FLISR solution is minimal, potentially leading to a substantial reduction in the delay from fault detection and resolution when complete human intervention is required, and therefore a significant saving in terms of penalties incurred by CMLs.

### Relevance to the DSO

This is significant as looking to the DSO’s performance reports, in 2019 service loss stood at 87.47 CML per customer (excluding storm events), resulting in a total financial penalty of €2.79m ^21^. Within certain EU territories, the relevant energy regulator imposes financial penalties on the DSO for the number of CMLs or CIs accrued per customer. In relation to Ireland, such regulatory policies are enforced by the Commission for Regulation of Utilities (CRU). For the Irish DSO this means that fines begin to accumulate once a fault event breaches an amount of CMLs that exceeds three minutes. With this in mind then, it is not only vital for the DSO to resolve faults in a timely fashion for the sake of the customer, but also to reduce the financial implications to the DSO to as high a degree as is possible. In practice, due to the manual operation required to operate pole mounted switches discussed previously, it may take well beyond the three-minute threshold for a fault event to be detected and ultimately resolved on a distribution grid.

The potential benefits of a FLISR solution such as the one described in this paper is compounded further when taking the effects of climate change and extreme weather events and their effect on the distribution network into account ^1^. Such disastrous effects were demonstrated in Ireland in 2017 by the enormous negative impact caused by ex-hurricane Ophelia, which resulted in almost 400,000 customers without power and a total of 11.7 million customer hours lost (CHL) according to the DSO’s 2017 performance report ^22^. As climate change and the resulting weather events continue to increase in size and severity in the future, the need for intelligent, automated systems to be made available will inevitably increase, exemplified by the Irish DSO’s own plans to increase the self-healing capabilities of the distribution network by 2027 by introducing new smart technologies and systems ^23^. The FLISR solution and related research described in this paper has the potential to meet the needs of the DSO and with further development has the capability to benefit both the DSO and energy consumer by not only aiding operations in the here and now, but also hardening the distribution grid against future challenges.

### Potential improvements

While the FLISR solution algorithms were developed, tested and implemented using predefined test cases to validate the solution outputs, such validation could have been further enhanced with the use of a simulated environment. As the structure of the distribution network is known, it would be possible to create a software simulator which would generate simulated fault events that would then trigger the FLISR solution to generate outputs. This would enable another layer of validation before implementing the solution on the trial site. Additionally, such a simulator would also enable the capability to simulate distribution grids located in a number of diverse locations, offering insight into the potential benefits of the FLISR solution in locations with unique operational challenges or network configurations. Although the development and implementation of such a simulator was outside the scope of work for this particular piece of research, further iterations would greatly benefit from its introduction.

**Figure 11.**
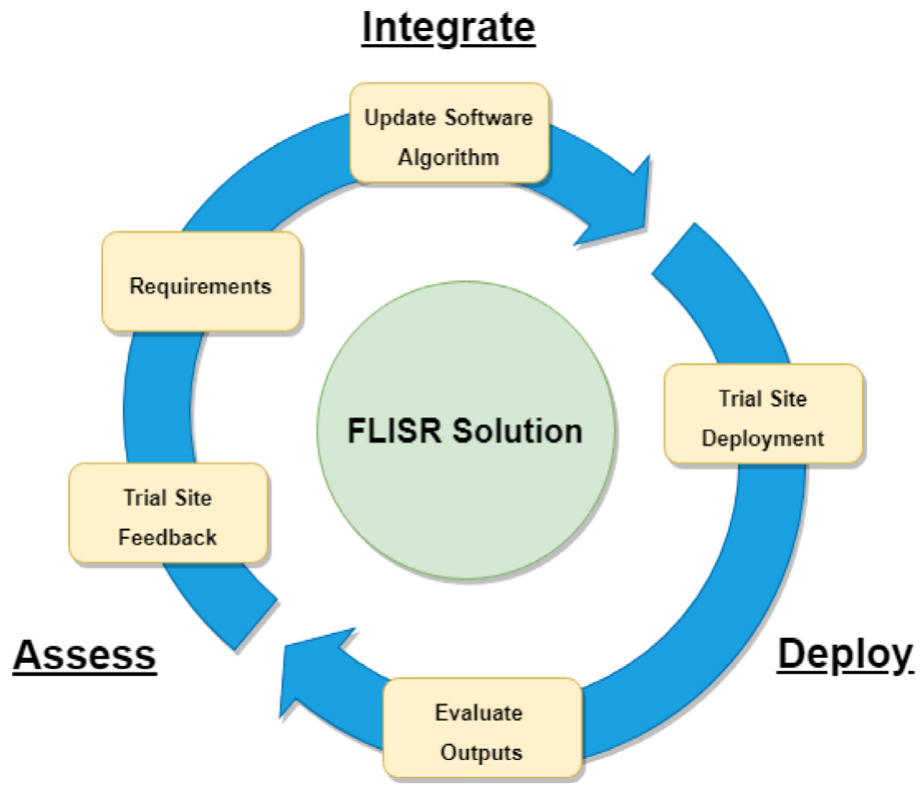
FLISR solution iterative development model

In addition, working closely with the DSO over a period of time to validate the algorithm outputs, would allow for greater optimisation of the algorithm through leveraging the expertise of operation staff. The feedback gathered could be utilised to ensure edge cases are identified and optimal, accurate restoration steps are generated for a wider range of network configurations. In this way, development of subsequent versions of the algorithm would take an iterative approach, directly applying inputs from the stakeholders to continually improve the algorithm outputs as proposed in Figure 11.

Furthermore, while the output of the FLISR solution in the form of an email report was one specifically requested by the DSO, with further enhancements to the algorithm, it would be possible to enable bi-directional communication with the pole-mounted switches, therefore enacting restoration steps in an automated fashion. With the aid of a simulator as described above, such a solution may be rigorously tested in a simulated environment, which may go some way toward mitigating the concerns of deploying a fully automated solution to a live grid. An alternative proposal would be to develop a hybrid solution, whereby restoration steps are emailed to a CRO, who would then validate the restoration steps and give permission for the FLISR solution to enact the restoration steps if the result is optimal.

## Conclusions

In this paper we have discussed how the DSO’s research on the best steps to isolate and resolve faults on part of an active distribution grid informed the development, implementation and testing of a novel software-driven FLISR solution. The proposed solution utilizes modern software and communication technologies in conjunction with existing grid models and pole-mounted devices on the trial site to provide system awareness, monitoring of fault events, and the generation of recommended steps to isolate and restore service, communicated to DSO system operators for enactment. From the results gathered, the delay in fault detection and reporting is minimal, leading to a potential reduction of the negative financial impact of CMLs as a result of fault events. The proposed FLISR solution could be enhanced further with the aid of network operators, validating the FLISR recommendations and suggesting changes to FLISR reporting. With such validation in place and further development, an intelligent, fully autonomous version of the FLISR solution could be realised, enabling bi-directional control of pole-mounted devices, thereby increasing the solutions impact-mitigation capability, further reducing the delay in fault resolution. Given the increasing disruption to the grid by climate change and the introduction of RES and distributed energy resources, the need for solutions that lever existing DSO data streams is apparent. The application of back-bone services and communication technologies explored in this research can be further leveraged for other use-cases in the energy space to meet this need, such as ancillary services for grid stability and interactions between the energy grid and market to facilitate intelligent energy flexibility.

## Data Availability

Zenodo: dLeniston/flisr-solution-paper-data: Version 1.0.0. https://doi.org/10.5281/zenodo.5667451 This repository contains the following data and resources: E11_circuit_decision_tree.xlsx (decision tree for E11 circuit which informed the development of the FLISR solution algorithm, it represents graphically the conditions under which certain fault cases occur) E11_circuit_CIM.json (Common Information Model (CIM) representation of the E11 circuit, one of the lines on which the solution was trialled. This file is written in JavaScript Object Notation (JSON) format and contains metadata relating to nodes on this circuit and their location relative to other assets. These models were utilised to provide system awareness in terms of where pole mounted switches are located relative to one another to enable the FLISR solution to produce meaningful outputs) E11_circuit_sample_fault_data.csv (Sample data set of fault readings for the E11 circuit is provided in a comma-separated values (CSV) file for investigation or importing into a time series database) E11_circuit_test_case_seeds (Contains individual files to seed a time series database with fault data for each fault case scenario as described in the paper) Data are available under the terms of the Creative Commons Zero "No rights reserved" data waiver (CC0 1.0 Public domain dedication). The underlying data outlined relates to one of the circuits described in the paper and was provided by the Irish DSO. As the full trial site grid model contains information relating to real, live grid assets and customer details, it has not been made publicly available as part of this paper. Further information, including contact information to request additional data directly from the DSO, is available at the following resource: ESB Networks: SOGNO H2020 Project Close-Out Report dleniston-tssg, & Darren Leniston. (2021). dLeniston/flisr-solution-paper-data: Version 1.0.0 (v1.0.0). Zenodo. https://doi.org/10.5281/zenodo.5667451
