# Supplementary material for: Implementation of a software defined FLISR solution on an active distribution grid
Source: Open Res Eur. 2022 Sep 20;1:142. Originally published 2021 Nov 29. [Version 2] doi: 10.12688/openreseurope.14115.2 (PMC10445810; doi:10.12688/openreseurope.14115.2)
Supplement: Peer review history

# Peer review: Reviewer response for version 2

**DOI:** 10.21956/openreseurope.16351.r30127

**Published:** 2022-09-30

**Authors:** Peter Watson

**Affiliations:** 1 Department of Civil & Environmental Engineering, University of Connecticut, Storrs, CT, USA

Overall, the revised version of this article is a great improvement over the original submission. It places itself within the current literature much more clearly, and explicitly discusses some of the advantages and disadvantages of their methodology.  While I have several remaining comments about the presentation of this work, it is particularly interesting because of the potential implications for how DSOs may implement FLISR solutions in the future.  

 Itemized Minor Comments:

1. The first mention of FLISR in the introduction should explain the meaning of the acronym. 
2. The structure of the introduction could be reorganized to improve readability. In the current draft elements of motivation, background, literature review, and description of this study are mixed together.  If these ideas were more clearly delineated it would be easier for readers to understand the current state-of-the-art, current gaps in implementation and understanding, and how this work addresses those gaps.
3. My original concern about the scalability of this methodology was not about the implementation of the software, but about the development of decision trees used. Designing the decision trees as described appears to be a laborious, iterative, and manual process that requires significant engagement from the DSO's engineers, even for a small system involving three substations. But the United States, for example, has more 75,000 substations. And with the rapid changes that distributed energy resources (DER) will introduce, it is likely that more dynamic and less supervised model-based optimization frameworks could be implemented more broadly to reduce the number of customers affected by unplanned power outages, like what is being developed in some advanced distribution management systems (ADMS).

Is the study design appropriate and does the work have academic merit?

Yes

Is the work clearly and accurately presented and does it cite the current literature?

Partly

If applicable, is the statistical analysis and its interpretation appropriate?

Not applicable

Are all the source data underlying the results available to ensure full reproducibility?

Yes

Are the conclusions drawn adequately supported by the results?

Yes

Are sufficient details of methods and analysis provided to allow replication by others?

Yes

Reviewer Expertise:

Power Outage Prediction, Data Science, Impact Modeling

I confirm that I have read this submission and believe that I have an appropriate level of expertise to confirm that it is of an acceptable scientific standard.

# Peer review: Reviewer response for version 2

**DOI:** 10.21956/openreseurope.16351.r30128

**Published:** 2022-09-22

**Authors:** Liu Xinyu

**Affiliations:** 1 College of Electrical Engineering and Automation, Fuzhou University, Fuzhou, China

Authors have modified the manuscript and solved most of the problems. I have no further questions.

Is the study design appropriate and does the work have academic merit?

Partly

Is the work clearly and accurately presented and does it cite the current literature?

Partly

If applicable, is the statistical analysis and its interpretation appropriate?

Not applicable

Are all the source data underlying the results available to ensure full reproducibility?

Yes

Are the conclusions drawn adequately supported by the results?

Partly

Are sufficient details of methods and analysis provided to allow replication by others?

Yes

Reviewer Expertise:

Fault diagnosis of power lines; Artificial intelligence; Deep learning

I confirm that I have read this submission and believe that I have an appropriate level of expertise to confirm that it is of an acceptable scientific standard.

# Peer review: Reviewer response for version 1

**DOI:** 10.21956/openreseurope.15218.r29160

**Published:** 2022-05-06

**Authors:** Peter Watson

**Affiliations:** 1 Department of Civil & Environmental Engineering, University of Connecticut, Storrs, CT, USA

This is a nice study of FLISR in application. Overall, I thought it was clearly written, and particularly appreciated the practical insights generated from deploying FLISR solution at a DSO. However, in my opinion there remains some room for improvements to this draft. Specifically:

 1) The literature review in the introduction is at a rather high level. How does this study compare to similar attempts to put FLISR into practice? There is some other work in this field which could help the reader appreciate this work in that context. For example, the US Department of Energy sponsored similar projects that are reported on here: https://openreseurope.s3.amazonaws.com/supplementary/14115/300a4714-32b1-4d8e-b561-3f09699fdc70.pdf

 2) Could the authors comment on the scalability of their approach? The power grid is arguably the largest and most complex engineered system on the planet, and with current innovations in the industry, the complexity is set to only increase further. Yet, in the described solution, they describe a process that relies on the manual analysis of utility experts to determine the fault states in the one circuit that's analyzed here. How can this solution be scaled in a way the significantly improves the reliability of power for most customers?

 3) Because of the lack of seconds in the 'Email report received' column, Table 4 doesn't seem to provide much information. 

 4) I'd like to second the suggestion that the authors presented about implementing this algorithm in a power system simulator. While the practical insights generated from putting this algorithm into practice are quite interesting, testing in a simulator would allow them to generate more data points, and allow for more rigorous testing of their algorithm with additional circuit designs.

 5) While the term 'self-healing' exists in the literature, I would question whether or not it is an appropriate term to use for FSLIR in the context of storm-related damages. FSLIR does not actually fix the faults in the power grid, but rather reroutes power around the faults to mitigate their impacts. Repair crews would still need to be deployed to manually repair all storm-related damages. Perhaps 'impact mitigation' would be a more appropriate term, because the process of healing the grid after a storm remains manual.

Is the study design appropriate and does the work have academic merit?

Yes

Is the work clearly and accurately presented and does it cite the current literature?

Partly

If applicable, is the statistical analysis and its interpretation appropriate?

Not applicable

Are all the source data underlying the results available to ensure full reproducibility?

Yes

Are the conclusions drawn adequately supported by the results?

Yes

Are sufficient details of methods and analysis provided to allow replication by others?

Yes

Reviewer Expertise:

Power Outage Prediction, Data Science, Impact Modeling

I confirm that I have read this submission and believe that I have an appropriate level of expertise to confirm that it is of an acceptable scientific standard, however I have significant reservations, as outlined above.

# Peer review: Reviewer response for version 1

**DOI:** 10.21956/openreseurope.15218.r28529

**Published:** 2022-02-16

**Authors:** Liu Xinyu

**Affiliations:** 1 College of Electrical Engineering and Automation, Fuzhou University, Fuzhou, China

Authors used the data from existing pole-mounted devices for automatic fault location and power restoration. The topic is actual and concerns the electrical companies.

 However, there are several points that should be taken into consideration.

 1. The proposed FLISR algorithm seems like an exhaustive strategy. How to design the recommended restoration steps when giving a set of device status? In other words, how to ensure the output recommended restoration steps are optimal? Does it have a standard?

 2. It is suggested to introduce the definition of hardware-driven and software-driven solutions. It is hard to follow that the software also needs the hardware (sensors or smart devices) to collect data.

 3. It is suggested to supplement some related works with references that also leverage the installed pole-mounted devices for automatic fault location and power restoration.

 4. A number of acronyms are present and a sort of nomenclature would be helpful for easier following.

 5. Sub-section titles in Methods are suggested to be modified to capture the core idea of the sub-section.

 6. The result does not reflect the accuracy of fault location.

Is the study design appropriate and does the work have academic merit?

Partly

Is the work clearly and accurately presented and does it cite the current literature?

Partly

If applicable, is the statistical analysis and its interpretation appropriate?

Not applicable

Are all the source data underlying the results available to ensure full reproducibility?

Yes

Are the conclusions drawn adequately supported by the results?

Partly

Are sufficient details of methods and analysis provided to allow replication by others?

Yes

Reviewer Expertise:

Fault diagnosis of power lines; Artificial intelligence; Deep learning

I confirm that I have read this submission and believe that I have an appropriate level of expertise to confirm that it is of an acceptable scientific standard, however I have significant reservations, as outlined above.
